# Síndrome de desgaste profesional, índice de masa corporal y otros factores asociados con la labor de profesores de educación física de Ibagué, Colombia

**DOI:** 10.7705/biomedica.4282

**Published:** 2019-09-01

**Authors:** Felipe Augusto Reyes-Oyola, Constanza Palomino-Devia

**Affiliations:** 1 Programa de Licenciatura en Pedagogía Infantil, Corporación Universitaria Minuto de Dios, Ibagué, Colombia Corporación Universitaria Minuto de Dios Programa de Licenciatura en Pedagogía Infantil Corporación Universitaria Minuto de Dios Ibagué Colombia; 2 Programa de Licenciatura en Educación Física, Facultad de Ciencias de la Educación, Universidad del Tolima, Ibagué, Colombia Universidad del Tolima Programa de Licenciatura en Educación Física Facultad de Ciencias de la Educación Universidad del Tolima Ibagué Colombia; 3 Facultad de Ciencias de la Educación, Universidad del Tolima, Ibagué, Colombia Universidad del Tolima Facultad de Ciencias de la Educación Universidad del Tolima Ibagué Colombia

**Keywords:** agotamiento profesional, síndrome de desgaste profesional, docentes, educación y entrenamiento físico, índice de masa corporal, grupos de edad, sexo, Burnout, professional, faculty, physical education and training, body mass index, age, sex

## Abstract

**Introducción.:**

El síndrome de desgaste profesional en profesores ha ido en aumento durante las últimas décadas y ha suscitado interés por su estudio.

**Objetivo.:**

Determinar los grados del síndrome de desgaste profesional y su asociación con otros factores de los profesores de educación física de las instituciones educativas del municipio de Ibagué.

**Materiales y métodos.:**

Se hizo un estudio descriptivo y transversal de 111 docentes de educación física de los colegios de Ibagué, con edades entre los 26 y los 65 años. Las variables sociodemográficas incluyeron el sexo y la edad, y se analizaron las variables propias del síndrome. La información se recolectó utilizando la versión para profesionales de la educación del ‘Cuestionario para la evaluación del síndrome de quemarse (sic) con el trabajo’ (CESQT-PE).

**Resultados.:**

Los profesores de educación física presentaron niveles bajos del síndrome; 22 docentes (19,8 %) presentaron niveles elevados y de estos, 15 respondían al perfil 1 (síndrome sin sentimientos de culpa) y siete al perfil 2 (síndrome con sentimientos de culpa). En los hombres la prevalencia del síndrome fue mayor, en tanto que en las mujeres las cifras fueron mayores en las dimensiones de desgaste físico y emocional, indolencia y sentimientos de culpa.

**Conclusiones.:**

Es necesario diseñar e implementar programas de formación orientados a explicar qué es el síndrome de desgaste profesional, cómo y por qué aparece, cómo evoluciona y cuáles son sus síntomas, así como estrategias de prevención e intervención individual que incluyan técnicas de relajación física y de control respiratorio.

## Abstract (en)

**Introduction:**

The burnout syndrome in teachers has been increasing during the last decades, which explains its becoming a very important study area.

**Objective:**

To determine the levels of the burnout syndrome and its relation with other factors among the physical education teachers in some schools of Ibagué.

**Materials and methods:**

We conducted a cross-descriptive correlational study among 111 physical education teachers from Ibagué with ages ranging from 26 to 65 years. We included gender and age as the sociodemographic variables, as well as the burnout syndrome-related variables. For data collection, we used a questionnaire in Spanish for the evaluation of the burnout syndrome specifically among teaching professionals.

**Results:**

The physical education teachers interviewed had low levels of burnout syndrome; 22 teachers (19.8%) had high levels and 15 of these responded to profile 1 (burnout syndrome without feelings of guilt) and 7 to profile 2 (burnout syndrome with feelings of guilt). Burnout syndrome prevalence was higher in men while in women the prevalence of physical and emotional exhaustion, indolence and negative attitudes, and feelings of guilt was higher.

**Conclusions:**

It is necessary to design and implement training programs aimed at explaining what burnout syndrome is, how and why it appears, its progress and symptoms, as well as strategies of individual prevention and intervention such as physical relaxation and respiratory control techniques.

El síndrome de desgaste profesional (*burnout syndrome*), característico de los profesionales de servicios de ayuda o de servicios humanos, se caracteriza por un estado de agotamiento como consecuencia del trabajo intenso, en el que se descuidan las necesidades propias. Las personas que lo padecen se agotan por su abnegada dedicación al trabajo ^1^. En este sentido, para satisfacer las exigencias de su trabajo y cumplir con sus obligaciones laborales, los individuos se exigen y se esfuerzan más allá de sus posibilidades, pasan sobre sus propios intereses y capacidades, y pagan un elevado precio para incrementar su rendimiento.

El síndrome de desgaste profesional en el profesorado ha ido en aumento durante las últimas décadas. En este contexto, en un estudio en el que se analizó el síndrome en una muestra amplia de profesionales de distintos ámbitos laborales (n=3.758), se encontró que los docentes están entre los que más se ven afectados por él, comparados con los de otras ramas, como los gerentes y directivos, los psicoterapeutas y trabajadores sociales, los médicos y dentistas, los enfermeros, las amas de casa y los trabajadores. En los profesores de ambos sexos se encontró un mayor agotamiento emocional y un menor logro personal, y se registraron en mayor proporción actitudes y comportamientos negativos hacia las demás personas ^2^.

La educación, específicamente la labor docente, es una de las profesiones en las que predomina un continuo contacto social con otras personas; por ello, su ejercicio implica mayores posibilidades de desarrollar el síndrome de desgaste profesional, con las consecuencias nocivas previsibles para la calidad de la enseñanza. El síndrome afecta a los profesores de todas las áreas de formación escolar sin excepción y desencadena un proceso de desgaste profesional caracterizado por un estado de frialdad, alienación, cinismo y apatía, que evidentemente puede perjudicar el clima organizacional y social del aula, así como la calidad de la enseñanza, además de incidir en el aumento del ausentismo laboral ^3^. Asimismo, los profesores agotados entregan menos información a sus alumnos y no reconocen sus logros, aceptan en menor medida sus ideas e interactúan con menor frecuencia con ellos, son menos simpáticos en su lugar de trabajo, presentan una menor tolerancia a las interrupciones durante las clases, preparan ineficazmente el material de enseñanza, y su nivel de entrega y dedicación profesional es menor; es decir, se sienten alienados con respecto a su trabajo ^4^.

El síndrome no solo afecta la calidad de la enseñanza, sino que también perjudica la salud de los docentes en todas sus dimensiones. Un profesor no corre el riesgo de caerse de un andamio, ni se ve afectado por la silicosis, pero su entorno también supone un riesgo para su salud física y, sobre todo, mental ^5^.

Entre las variables sociodemográficas del profesorado asociadas con este síndrome sobresalen el sexo y la edad. En estudios sobre profesores griegos ^6^, brasileros ^7^, españoles ^8^, italianos ^9^ y venezolanos ^10^, se determinó la relación entre el estado del síndrome de desgaste profesional y la edad. Asimismo, en un estudio en docentes argentinos se estableció la relación entre las dimensiones del síndrome y la adiposidad con base en el índice de masa corporal (IMC), y se establecieron relaciones significativas entre este y la dimensión de la realización profesional, lo que indicó que, cuando un sujeto se siente profesionalmente realizado, se preocupa más por su salud, incluido el mantenimiento de un peso adecuado ^11^.

La naturaleza de la enseñanza de la educación física puede ocasionar en los docentes grados moderados o elevados del síndrome. Los docentes de educación física se encuentran constantemente expuestos a factores laborales estresantes debido a las características de la asignatura, el constante movimiento del alumnado en la clase, el bajo estatus social entre el colectivo docente, y la falta de instalaciones y equipos adecuados o a sus malas condiciones ^12^, así como a la escasez del material didáctico disponible. Todo esto origina la disminución de los grados de motivación y la aparición del agotamiento físico y mental entre los profesionales ^13^.

En este contexto, y con base en las variables determinantes que explican la etiología del síndrome de desgaste profesional, el objetivo central del presente estudio fue determinar sus su intensidad y su relación con el IMC en los profesores de educación física de instituciones educativas del municipio de Ibagué, así como comparar los grados del síndrome entre los grupos por sexo y edad.

## Materiales y métodos

El estudio tuvo un enfoque cuantitativo, no experimental, de diseño transversal y descriptivo ^14^. La población incluyó a los profesores del área de educación física de 61 instituciones educativas del municipio de Ibagué -55 establecimientos oficiales y seis privados-, distribuidas en las 13 comunas y seis corregimientos de la ciudad ^15^.

En el estudio se incluyeron a todos los docentes de educación física de Ibagué que laboraban en básica primaria, básica secundaria y media. El 76,6 % correspondía a hombres (n=85) y el 23,4 % a mujeres (n=26); las edades de los participantes estaban entre los 26 y los 65 años (45,1±10,2); el 84,7 % de ellos trabajaba en el sector oficial y el 15,3 % en el sector privado. Los profesores debían ser licenciados en el área de Educación Física y no se incluyó a quienes tenían a cargo la asignatura de educación física pero eran especializados en otra área educativa. Todos los docentes seleccionados accedieron a participar en el estudio.

La información se recolectó en tres etapas. En la primera, se registró la identificación general del docente, su edad, su sexo y la institución educativa a la cual pertenecía. En la segunda etapa, se determinó la masa corporal utilizando una báscula Fitscan Body Monitor™ (BF-679F) (Tanita) y la estatura con un tallímetro mecánico para niños y adultos (ref. 216, seca), siguiendo los protocolos internacionales para la valoración antropométrica de la *International Society for the Advancement of Kinanthropometry* (ISAK) ^16^.

En la tercera etapa, se empleó el ‘Cuestionario para la evaluación del síndrome de quemarse (sic) con el trabajo’, en su versión para profesionales de la educación (CESQT-PE) ^17^, el cual consta de 20 preguntas cerradas con escalonamiento de tipo Likert. Las opciones de respuesta son: 0 (nunca), 1 (raramente: algunas veces al año), 2 (a veces: algunas veces al mes), 3 (frecuentemente: algunas veces por semana) y 4 (muy frecuentemente: todos los días).

Este cuestionario está conformado por las dimensiones de ‘ilusión por el trabajo’ (5 ítems), ‘desgaste psíquico’ (4 ítems), ‘indolencia’ (6 ítems) y ‘culpa’ (5 ítems). La ‘ilusión por el trabajo’ es el deseo del individuo de alcanzar las metas laborales porque supone una fuente de placer personal; el ‘desgaste psíquico’ es la aparición del agotamiento emocional y físico por el trato diario con personas que tienen problemas o los causan; la ‘indolencia’ es la aparición de actitudes negativas de indiferencia y cinismo hacia los receptores de la labor que se desempeña, y la ‘culpa’ aparece a causa de los comportamientos y las actitudes negativas en el trabajo, en especial, hacia las personas con las que se establecen relaciones laborales ^17^.

Cuando las puntuaciones en la dimensión de la ‘ilusión por el trabajo’ son bajas y las del ‘desgaste psíquico e indolencia’ y de ‘culpa’ son altas, se considera que hay grados elevados del síndrome ^17^. Para determinar los grados altos y bajos del síndrome, se utilizó el procedimiento de Shiron, en el cual se consideran altas las puntuaciones de aquellos participantes con un promedio igual o superior a 2 (algunas veces al mes) en las dimensiones de ‘desgaste psíquico’, ‘indolencia’ y ‘culpa’ y con uno inferior a 2 (algunas veces al mes) en la dimensión de ‘ilusión por el trabajo’ ^18^.

Para categorizar los niveles del síndrome como ‘perfil 1’ o ‘perfil 2’, además de considerar las cuatro dimensiones mencionadas, el CESQT también permite una puntuación total del síndrome, la cual es el resultado de promediar las puntuaciones en los 15 ítems que conforman las dimensiones de ‘ilusión por el trabajo’, ‘desgaste psíquico’ e ‘indolencia’. Los ítems de la dimensión de ‘culpa’ no contribuyen al cálculo de la puntuación del síndrome, ya que esta tiene como función distinguir entre dos perfiles diferenciados del síndrome: el perfil 1 (sin niveles altos de culpa) y el perfil 2 (con grados altos de culpa) ^19^.

Más explícitamente, el perfil 1 corresponde a individuos que sufren estrés moderado relacionado con el trabajo, y se caracterizan por demostrar poco entusiasmo para hacerlo y presentar altos niveles de agotamiento psicológico e indolencia, pero que, a pesar de tales problemas, todavía son capaces de hacer su trabajo y no experimentan fuertes sentimientos de culpa. Las personas que responden al perfil 2 son aquellas afectadas con mayor intensidad por los síntomas, pues no pueden hacer su trabajo correctamente y ello las lleva a desarrollar sentimientos de culpa ^20^^).^

La información recolectada se analizó con el paquete estadístico IBM- SPSS™, versión 23, mediante un análisis descriptivo que arrojó la media, la desviación estándar, la frecuencia absoluta y la frecuencia relativa expresadas en porcentajes, según el caso. Se obtuvieron los intervalos de confianza de las medias de 95 % como indicador de fiabilidad. Para las comparaciones entre grupos, se usó un modelo lineal general multivariante. La significación estadística se estableció con un valor de p<0,05.

Para efectuar la comparación de medias de las dimensiones del síndrome de desgaste profesional según los grupos de edad, se efectuó un análisis de varianza (ANOVA) de un factor. Para comprobar la existencia de diferencias entre las medias de las variables analizadas, se utilizó la prueba t de Student para dos muestras independientes. Por último, se determinó la relación entre las variables del síndrome y la edad y el IMC empleando el coeficiente de correlación de Pearson (*r*).

El estudio se ajustó a los parámetros éticos, como el derecho a no recibir ofensas, a la determinación personal, a la privacidad (en cuanto a los sentimientos, actitudes, valores, información personal, etc.) y a la conservación de la integridad personal.

## Resultados

En el cuadro 1 se presenta el índice de masa corporal de los profesores participantes clasificado en bajo peso, peso normal, sobrepeso, obesidad I, obesidad II y obesidad III ^21^. Según los resultados obtenidos, el 60,4 % de ellos tenía sobrepeso y obesidad, y el 38,7 %, peso normal. La distribución de los niveles del IMC por sexo también evidenció la prevalencia de sobrepeso en hombres (54,1 %) y en mujeres (57,7 %), y también, por grupos etarios: en menores de 40 años (54,1 %), de 40 a 50 años (58,3 %) y más de 50 años (52,6 %). Además, los valores del IMC en los hombres (25,82 kg/m2) fueron mayores que los de las mujeres (25,22 kg/m2), aunque sin diferencias estadísticamente significativas (p>0,05).

**Cuadro 1:** Valores del índice de masa corporal, distribución por sexo y grupos etarios

Niveles de IMC^£^	Bajo peso (%)	Peso normal (%)	Sobrepeso (%)	Obesidad (%)
Todos (n=111)	0,9	38,7	55	5,4
Sexo				
Femenino (n=26)	0	38,5	57,7	3,8
Masculino (n=85)	1,2	38,8	54,1	5,4
Grupos etarios años				
<40 (n=37)	0	43,2	54,1	2,7
40 a 50 (n=36)	2,8	33,3	58,3	5,6
>50 (n=38)	0	39,5	52,6	7,9

£ Índice de masa corporal

En el cuadro 2 se presentan los grados del síndrome de desgaste profesional de los participantes. Los resultados reflejan que el 80,2 % (n=89) de ellos presentaba grados bajos del síndrome y, el 19,8 % (n=22), grados altos.

**Cuadro 2:** Frecuencia y porcentaje de profesores de educación física con grados altos y bajos del síndrome de desgaste profesional

SDPЖ y dimensiones	Niveles altos (≥2)		Niveles bajos (<2)		Total	
	Frecuencia absoluta	Frecuencia relativa (%)	Frecuencia absoluta	Frecuencia relativa (%)		
SDP	22	19,8	89	80,2	111	100
	Perfil 1	15	68,2	-	-	15	13,5
Perfil 2		7	31,8	-	-	7	6,3
	Ilusión por el trabajo	109	98,2	2	1,8	111	100
Indolencia		27	24,3	84	75,7	111	100
	Desgaste psíquico	38	34,2	63	65,8	111	100
Culpa		19	17,1	92	82,9	111	100

Ж Síndrome de desgaste profesional

De los 22 profesores con grados altos del síndrome, 15 (68,2 %) respondían al perfil 1 (sin sentimientos de culpa) y siete (31,8 %) al perfil 2 (con sentimientos de culpa). Los profesores con altos grados en el perfil 1 correspondían al 13,5 % del total y, en el perfil 2, al 6,3 %. Además, los análisis descriptivos de cada dimensión del síndrome evidenciaron que el 98,2 % de los profesores presentaba grados altos en la dimensión de ‘ilusión por el trabajo’, es decir, se sentían motivados por su trabajo, percibiéndolo como atractivo y agradable. En cuanto a la dimensión de ‘indolencia’, el 24,32 % de los profesores presentó grados altos, evidenciando actitudes negativas como cinismo e indiferencia frente a los demás integrantes de la comunidad educativa; por otro lado, el 75,68 % presentó promedios bajos en esta dimensión.

Con respecto a la dimensión de ‘desgaste psíquico’, el 65,77 % de los profesores obtuvo grados bajos, en tanto que el 34,23 % registró niveles altos de “agotamiento”, tanto físico como emocional. Por último, en la dimensión de “culpa” el 82,88 % de los educadores presentó grados bajos, y el restante 17,12 %, promedios altos, lo que refleja que estos profesores pueden sentir mucha preocupación por los daños ocasionados, enojo contra sí mismos o temor de ser descubiertos.

En el cuadro 3 se presentan los resultados del análisis estadístico descriptivo y la comparación de los grupos por sexo. Los resultados evidenciaron que los hombres tuvieron mayores grados de ilusión y motivación hacia su profesión en comparación con las mujeres, aunque sin diferencias estadísticamente significativas (p>0,05). Las mujeres tuvieron grados más altos en las dimensiones de ‘desgaste psíquico’, ‘indolencia’ o ‘conductas negativas y sentimientos de culpa’, con diferencias significativas en la dimensión de ‘desgaste psíquico’ (p<0,05).

**Cuadro 3:** Niveles de las dimensiones del síndrome de desgaste profesional, comparación según sexo

Dimensión	Todos (n=111)		Mujeres (n=26)		Hombres (n=85)		p*
	X ± DE	IC^95% ¥^	X ± DE	IC^95% ¥^	X ± DE	IC^95% ¥^	
Ilusión por el trabajo	3,65±0,5	3,56-3,75	3,56±0,49	3,37-3,76	3,68±0,50	3,57-3,79	0,319
Desgaste psíquico	1,22±1,03	1,02-1,41	1,61±1,02	1,20-2,02	1,10±1	0,88-1,32	0,026
Indolencia	1,1±0,81	0,94-1,25	1,24±0,88	0,88-1,60	1,05±0,79	0,88-1,23	0,315
Culpa	0,94±0,73	0,80-1,08	0,96±0,81	0,63-1,28	0,93±0,71	0,78-1,09	0,891

X ± DE: promedio ± deviación estándar

¥ Media ± desviación estándar e intervalo de confianza de 95 %

* Nivel de significación entre promedios por sexo

En el cuadro 4 se presentan los resultados de la comparación entre los grupos de edad en las dimensiones del síndrome. En la dimensión de ‘ilusión por el trabajo’, se observó que con la edad aumentaban los valores en la motivación por la labor docente. La indolencia disminuyó con la edad y el desgaste psíquico fue mayor en los docentes de menos de 30 años y en el grupo de 30 a 40 años. Los sentimientos de culpa fueron mayores en el grupo de los menores de 30 años. No se encontraron diferencias estadísticamente significativas en las comparaciones de los grupos de edad en las dimensiones del síndrome (p>0,05).

**Cuadro 4:** Comparación entre grupos de edad en años de los profesores de educación física de Ibagué

Dimensión	<30 años (n=8)		30-40 años (n=32)		41-50 años (n=33)		>50 años (n=38)		p*
	X ± DE	IC^95% ¥^	X ± DE	IC^95% ¥^	X ± DE	IC^95% ¥^	X ± DE	IC^95% ¥^	
Ilusión por el trabajo	3,45±0,69	2,87-4,02	3,61±0,47	3,44-3,78	3,69±0,57	3,48-3,89	3,7±0,42	3,55-3,84	0,587
	Desgaste psíquico	1,40±1,01	0,55-2,25	1,48±0,96	1,13-1,83	1,21±0,96	0,87-1,56	0,96±1,11	0,6-1,33	0,199
Indolencia		1,54±0,59	1,04-2,03	1,23±1,01	0,87-1,06	1,01±0,65	0,77-1,24	0,97±0,77	0,71-1,22	0,206
	Culpa	1,05±0,93	0,27-1,82	0,98±0,79	0,7-1,27	1,01±0,67	0,77-1,24	0,82±0,69	0,59-1,05	0,677

X ± DE: promedio ± deviación estándar

¥ Media ± desviación estándar e intervalo de confianza de 95 % (IC95%)

*Nivel de significación entre promedios por grupos de edad

En el cuadro 5 se registran los resultados de la prueba estadística del coeficiente de Pearson para determinar las posibles relaciones entre las dimensiones del síndrome y la edad y el IMC. Solo se evidenció una relación entre la edad y el síndrome, específicamente una correlación negativa débil entre la edad y la dimensión de ‘desgaste psíquico’ (r=-0,214; p=0,024), es decir, a medida que aumentaba la edad, disminuyeron los grados de agotamiento emocional y físico en los profesores. El IMC no mostró ninguna relación con las dimensiones del síndrome (p>0,05).

**Cuadro 5:** Correlación entre las variables del síndrome de desgaste profesional y la edad de los profesores de educación física

Variables		Dimensiones del SDP^£^			
		Ilusión por el trabajo	Desgaste psíquico	Indolencia	Culpa
Edad	rЖ	0,086	-0,214*	-0,158	-0,106
	Nivel de significación	0,372	0,024	0,098	0,266
IMC^ϧ^	rЖ	-0,171	0,081	-0,048	0,026
	Nivel de significación	0,073	0,400	0,620	0,789

£ Síndrome de desgaste profesional

ϧ Índice de masa corporal

Ж Correlación de Pearson

* Correlación significativa= 0,05 (bilateral).

## Discusión

Los resultados del presente estudio permitieron determinar los grados del síndrome de desgaste profesional y del índice de masa corporal en los profesores de educación física de las instituciones educativas del municipio de Ibagué; además, se determinó la relación entre las dimensiones del síndrome, la edad y el IMC.

Los datos del cuadro 1 evidencian que un poco más del 60 % de los profesores tenían sobrepeso y obesidad, cifra preocupante debido al papel de los profesionales de esta área en la promoción de buenos hábitos y estilos de vida. Esta imagen corporal afectaría la llamada triada de empatía, puesto que en estos profesores la falta de adecuada apariencia física repercute en la poca o nula influencia de sus mensajes sobre la práctica del deporte y el ejercicio físico y la salud dirigidos a los estudiantes ^22^.

Al comparar el IMC de los docentes de Ibagué con el registrado en otros estudios, se evidenció que en 479 profesores mexicanos de escuelas de zonas rurales y urbanas los valores fueron ligeramente más elevados, con prevalencias de sobrepeso y obesidad del 64 %; la prevalencia de sobrepeso fue de 51 % en los hombres y de 36 % en mujeres, en tanto que la obesidad afectó al 20 % de los maestros y al 22 % de las maestras ^23^. Por el contrario, en un estudio con 186 docentes de la provincia de Córdoba, Argentina, de edades entre los 23 y los 64 años, se reportó que el 78,5 % de ellos tenía un peso normal, en tanto que solo el 21,5 % tenía sobrepeso y obesidad ^11^; en cuanto a los niveles de IMC por sexo, se reportó un peso normal en 68,7 % de los hombres y en 86,4 % de las mujeres ^11^.

Los resultados reportados en el cuadro 2 indican grados bajos del síndrome en los participantes de Ibagué, lo que coincide con el estudio hecho en 106 docentes de educación física mexicanos, quienes registraron grados bajos en las dimensiones del síndrome de fatiga crónica ^24^. Asimismo, se hallaron bajos índices del síndrome en estudios sobre docentes de educación física de México ^24^^)^ y Brasil ^25^^-^^29^.

Se consultaron algunos estudios sobre docentes de educación física de los sectores oficial y privado, por ejemplo, en España ^8^^,^^26^, en los que no se analizaron los grados del síndrome según el sector laboral. Es el caso de un estudio sobre profesores de todas las áreas llevado a cabo en dos instituciones educativas formales, una privada y una pública, de Cali, en el cual el 15 % de los profesores del colegio público y el 22 % de los educadores del privado presentaba grados moderados del síndrome ^27^^).^

En el mismo cuadro 2 se evidencia que el 34,2 % de los docentes de educación física de Ibagué se sentía agotado tanto física como emocionalmente, lo que probablemente conlleva conductas y actitudes negativas de cinismo, indiferencia, empleo de malas palabras en contra de los estudiantes, otros profesores, directivos e, incluso, padres de familia, con los consecuentes remordimientos debido a sus malos comportamientos.

En este sentido, en un estudio de 94 docentes de educación física brasileros de la ciudad de Pelotas (Estado de Río Grande del Sur), se encontraron altos grados de agotamiento emocional (60,6 %) y de despersonalización (22,3 %) ^9^. En otro estudio sobre profesores del municipio de Piraquara (Estado de Paraná), se encontró que 13 individuos (37,14 %) presentaban valores elevados de desgaste psíquico, tres (8,57 %), de indolencia, y tres, de culpa ^25^.

En este sentido, resulta positivo que la mayoría de los maestros de educación física de Ibagué presentaron grados elevados de ilusión por el trabajo, es decir, se sentían motivados frente a su labor educativa, y lograban las metas y propósitos profesionales establecidos, lo que constituye una fuente de satisfacción personal. Datos semejantes se hallaron en un estudio de 357 docentes de educación física españoles, que presentaban altos valores en la motivación intrínseca y la eficacia profesional ^26^ y, en otro de profesores brasileros, el porcentaje (5,71 %) (n=2) de valores bajos en la subescala de ‘ilusión por el trabajo’ fue mínimo ^25^.

En el cuadro 3 se evidencia que las profesoras registraron cifras más elevadas en las dimensiones de ‘desgaste psíquico’, ‘indolencia’ y ‘culpa’ en comparación con los hombres; esto coincide con los estudios sobre docentes brasileros ^28^^,^^29^, portugueses ^30^ y franceses ^31^, en los cuales se encontró que las profesoras registraban niveles más altos de agotamiento emocional y despersonalización. No obstante, en estos estudios las mujeres también reportaron un mayor sentimiento de realización personal, lo que no sucedió en el estudio de Ibagué, puesto que fueron los profesores quienes tuvieron mayores grados de ilusión por su trabajo. Asimismo, en un estudio de 219 profesores de educación física de escuelas secundarias italianas, se encontró que las mujeres tuvieron mayores puntuaciones en la dimensión de ‘agotamiento emocional’ y ‘baja realización personal’ que los hombres ^9^.

Los resultados que se presentan en el cuadro 4 evidencian que los profesores de educación física de Ibagué de 40 años o menos registraron las cifras más elevadas en las dimensiones de ‘desgaste psíquico’ e ‘indolencia’, resultados similares a los datos empíricos que indican que los profesores jóvenes se ven más afectados por el agotamiento emocional y físico y las actitudes negativas hacia los demás ^32^^-^^34^.

En el cuadro 5, se puede apreciar que la edad solo tuvo una correlación negativa débil con la dimensión de ‘desgaste psíquico’. Por el contrario, en un estudio de docentes venezolanos de educación física no se encontraron relaciones entre el grado del síndrome, sus dimensiones y la edad ^10^, y en uno de 217 profesores brasileros en el que se determinaron los niveles del síndrome y su asociación con variables demográficas (entre ellas, el sexo y la edad) y ocupacionales, no se encontró una asociación significativa (p>0,05) entre las dimensiones del síndrome y las variables demográficas ^35^.

En el presente estudio se logró determinar que la mayoría de los profesores de educación física participantes tenían sobrepeso y obesidad, con lo cual el riesgo de sufrir enfermedades cardiovasculares, diabetes, trastornos del aparato locomotor y algunos cánceres, se incrementa. Asimismo, presentaron grados bajos del síndrome, aunque hubo un número importante de aquellos con el perfil 1 que presentó grados altos del síndrome, en tanto que los de perfil 2 presentaron desgaste psíquico, indolencia y sentimientos de culpa. Solo en la dimensión de ‘desgaste psíquico’ se encontró una asociación significativa con factores como la edad.

En este sentido, deben implementarse intervenciones individuales y en el lugar de trabajo (cognitivas, conductuales, de relajación e integrales) para prevenir este síndrome ^36^^,^^37^. A partir de los resultados del estudio, puede inferirse que la mayoría de los profesores del área de educación física de Ibagué presentaban bajos grados del síndrome, aunque no deben descuidarse las estrategias para prevenirlo. 
